# Characterization of color variation in bamboo sheath of *Chimonobambusa hejiangensis* by UPLC-ESI-MS/MS and RNA sequencing

**DOI:** 10.1186/s12870-023-04494-3

**Published:** 2023-10-06

**Authors:** Lei Yu, Jinjun Yue, Yaxing Dai, Ling Zhang, Qiu Wang, Jinling Yuan

**Affiliations:** 1 Research Institute of Subtropical Forestry, Chinese Academy of Forestry, Fuyang District, Hangzhou, 311400 China; 2 Forestry and Bamboo Bureau of Changning County, Sichuan Province, 644300 China

**Keywords:** Flavonoid, Anthocyanin, Metabolomics, Transcriptome, *Chimonobambusa hejiangensis*

## Abstract

**Background:**

*Chimonobambusa hejiangensis* (*C.hejiangensis*) is a high-quality bamboo species native to China, known for its shoots that are a popular nutritional food. Three *C.hejiangensis* cultivars exhibit unique color variation in their shoot sheaths, however, the molecular mechanism behind this color change remains unclear.

**Methods:**

We investigated flavonoid accumulation in the three bamboo cultivar sheaths using metabolomics and transcriptomics.

**Results:**

UPLC-MS/MS identified 969 metabolites, with 187, 103, and 132 having differential accumulation in the yellow-sheath (YShe) vs. spot-sheath (SShe)/black-sheath (BShe) and SShe vs. BShe comparison groups. Flavonoids were the major metabolites that determined bamboo sheath color through differential accumulation of metabolites (DAMs) analysis. Additionally, there were 33 significantly differentially expressed flavonoid structural genes involved in the anthocyanin synthesis pathway based on transcriptome data. We conducted a KEGG analysis on DEGs and DAMs, revealing significant enrichment of phenylpropanoid and flavonoid biosynthetic pathways. Using gene co-expression network analysis, we identified nine structural genes and 29 transcription factors strongly linked to anthocyanin biosynthesis.

**Conclusion:**

We identified a comprehensive regulatory network for flavonoid biosynthesis which should improve our comprehension of the molecular mechanisms responsible for color variation and flavonoid biosynthesis in bamboo sheaths.

**Supplementary Information:**

The online version contains supplementary material available at 10.1186/s12870-023-04494-3.

## Background

China has extensive bamboo resources and a large bamboo industry, with a bamboo forest area of over 7 million hectares, and is the largest producer and exporter of bamboo shoots. *Chimonobambusa hejiangensis* C.D.Chu et C.S.Chao is a famous bamboo species of high economic value in domestic and foreign markets because its bamboo shoots taste crisp and have a beautiful color [1]. Bamboo shoots are consumed as high-quality vegetables because of their high protein, essential amino acids, crisp texture, fiber and low fat content [2, 3]. Bamboo shoot sheaths are an important bamboo organ, not only for bamboo species classification, but as the main protection and support organ of young bamboo shoots, and their important influence on bamboo shoot growth and quality [4, 5]. Bamboo sheath color directly affects bamboo shoot price and market competitiveness, and is one of the agronomic traits most sought after by bamboo farmers.

Flavonoids are essential plant secondary metabolites (PSMs) with high constituent content in bamboo leaves, and a wide range of applications in the food industry and traditional medicine [6]. Bamboo leaf extract has free radical scavenging, antioxidant, anti-inflammatory and antibacterial properties and its main functional components are phenolic acids and flavonoids, which are considered safe functional food additives [7–9]. Additionally, numerous studies have extracted flavonoids and phenolic acids from different structural bamboo parts such as shoots, culms [10], shoot sheaths [11], shavings extract [12] and fruits [13]. Flavonoids give plants a wide range of colors from light yellow to blue and improve their ability to resist biotic and abiotic stresses. Flavonoids are classified into six main groups, including flavanones, flavones, isoflavonoids, flavanols, flavonols, and anthocyanins. Flavonoid biosynthesis, catabolism and accumulation can lead to specific color development in plants, however, the underlying pigmentation regulatory mechanisms in bamboo shoot sheaths are still poorly understood.

Flavonoid synthesis starts from phenylalanine and goes through three stages: phenylalanine metabolism, flavonoid synthesis and anthocyanin synthesis. Stage I: phenylalanine is formed into 4-coumaroyl coenzyme A by three enzymatic reactions of phenylalanine lyase (PAL), cinnamic acid hydroxylase (C4H) and 4-coumaroyl coenzyme A ligase (4CL). Stage II: 4-coumaroyl coenzyme A is formed into dihydroflavonol by three enzymatic reactions of chalcone synthase (CHS), chalcone isomerase (CHI) and three enzymatic reactions of flavanone 3-hydroxylase (F3H) to form dihydroflavonol. The third stage is anthocyanosides synthesis, in which dihydroflavonol is hydroxylated by flavonoid 3’-hydroxylase (F3’H) and flavonoid 3’5’-hydroxylase (F3’5’H). They are then catalyzed by dihydroflavonol 4-reductase (DFR) to colorless anthocyanins, which are converted into colored anthocyanosides by anthocyanin synthase (ANS) and flavonoid 3-glucosyltransferase (3GT) action and stored in the vacuoles [14]. Researchers have identified 13 genes encoding enzymes (*PAL*, *C4H*, *4CL*, *CHS*, *CHI*, *FLS*, *F3′H*, *F3′5′H*, *DFR*, *ANS*, *LAR*, *IFS*, and *FNS*) related to the flavonoid metabolic pathway. In addition to these genes encoding enzymes involved in flavonoid biosynthesis pathways are several transcription factors (TFs), including R2R3-MYB, bHLH, WD40, WRKY, bZIP and MADS-box TFs that play a role in different branches of flavonoids biosynthesis [15]. Among these regulatory proteins, the R2R3-MYB, basic helix–loop–helix (bHLH) and WD40 proteins generally form an MBW(MYB-bHLH-WD40) transcriptional activation complex that regulates the transcription of genes encoding late anthocyanin biosynthesis (*DFR*, *LODX*, *ANR*) [16]. R2R3-MYB genes such as *MYB11*, *MYB12* and *MYB111* also independently regulate early anthocyanin biosynthesis genes (*CHS*, *CHI*, *F3H*, and *FLS*) [17]. Overall, flavonoid structural genes and transcriptional genes are involved in regulating secondary metabolite biosynthesis and color change, and the metabolic/transcriptional regulatory mechanism of bamboo shoot sheath color variation remains to be elucidated.

With the rapid development and wide application of high-throughput sequencing technologies, integrative genomic analyses such as metabolomics and transcriptomics have made it possible to accurately identify metabolites and associated genes for plant organ coloration [18]. We describe transcriptomic and metabolomic changes in three bamboo cultivars to investigate coloration mechanisms.

## Materials and methods

### Plant materials

We selected three bamboo cultivars (*C.hejiangensis*) with different sheath colors: yellow, spot, and black. To simplify the cultivar names, we abbreviated them as YShe for yellow-sheath cultivar ‘auratais’, SShe for spot-sheath cultivar ‘melagre’, and BShe for black-sheath cultivar ‘nigra’. Bamboo sheath samples were obtained from Fenlong Village, Shuiwei Town, Xuyong County, Sichuan Province, China (28.272064′N, 105.540995′E). Three bamboo cultivars (9 samples in total) were selected from three biological replicates, placed in liquid nitrogen containers, and stored in the laboratory at -80 °C. Bamboo sheaths from each material were randomly divided into three parts for metabolite extraction, RNA-Seq and qRT-PCR.

### Total anthocyanins analysis

The spectrophotometric pH difference method was employed to determine the overall bamboo sheath anthocyanin content [19]. The frozen samples were pulverized into powders in liquid nitrogen. One gram of powder with 10mL of 60% ethanol, was extracted in a 40 ℃ water bath for 60 min, and the extract was centrifuged at 4000 r/min for 15 min. One mL of the supernatant was added to buffer A (200 mM KCl and 200 mM HCl, pH 1.0) and buffer B (200 mM sodium acetate and 120 mM HCl, pH 4.5), each with a volume of 9 mL. Anthocyanin content was determined using the following formula: content (mg∙100 g^− 1^∙FW)=(A1-A2)×484.8 × 100 × 100/24,825, where A1/A2 is the absorbance at 510 nm of the supernatant collected in Buffer A/B solution. The molecular weight of cyanide-3-glucoside chloride is 484.8 and its molar absorbance at 510 nm is 24,825. Three independent experiments were conducted to determine the anthocyanin content of each sample.

### Metabolite profiling using UPLC-MS/MS

The YShe, SShe, and BShe samples were freeze-dried and then crushed using a mixer mill (MM 400, Retsch, Germany) with a zirconia bead for 1.5 min at 30 Hz. Next, 100 mg of sample powder was extracted in 1.0 mL of 70% aqueous methanol (v/v) at 4 ℃ overnight. After centrifugation at 12,000 rpm for 10 min, the extracts were filtered using a pore size of 0.22 μm (ANPEL, Shanghai, China) before UPLC-ESI-MS/MS analysis. The analytical conditions have been fully described by Li et al. [20]. We used ESI-triple Q-TRAP-MS to analyze the effluent, following the method described by Chen et al. [21]. A mixture of all sample extracts served as quality control (QC) for technical reproducibility. We quantified metabolites using multiple reaction monitoring mode (MRM) on a triple quadrupole mass spectrometer and analyzed UPLC-MS/MS data from bamboo sheath with Analyst 1.6.3 software (AB SCIEX, Ontario, Canada). To monitor technical reproducibility, we added a QC analysis after every 10 samples.

Metabolite qualitative and quantitative mass spectrometry analysis in project samples is based on the KEGG compound database [22], the MetWare database (MWDB), and multiple reaction monitoring (MRM). Metabolite identification relies on accurate metabolite mass, MS2 fragments, MS2 fragment isotope distribution, and retention time (RT) [23]. Using the company’s self-developed intelligent secondary spectrum matching method, we compare the secondary spectrum and metabolite RT in the project samples with the company’s database. The secondary spectrum and RT are intelligently matched one by one, with MS tolerance and MS2 tolerance both set at 20 ppm.

At Wuhan MetWare Biotechnology Co., Ltd., we followed standard procedures to identify and quantify the sample metabolites. We used the Metware (MWDB) and KEGG databases for metabolite annotation (Table S1). To ensure data normalization and homogeneity of variance, we log-transformed the original metabolite abundances. Subsequently, we conducted principal component analysis (PCA), hierarchical clustering analysis (HCA), and orthogonal projections to latent structures-discriminant analysis (OPLS-DA) using R software. We used the first OPLS-DA component to extract VIP values for all metabolites. To identify differentially accumulated metabolites among varieties (YShe vs. SShe, YShe vs. BShe, SShe vs. BShe), we compared only those with VIP ≥ 1, *p*-value < 0.05 and fold change ≥ 2 or fold change ≤ 0.5.

### RNA-seq analysis

We extracted total RNA from bamboo sheath YShe, SShe, and BShe group samples using a Trizol kit (Vazyme, Nanjing, China). RNA quality was assessed with a NanoPhotometer (IMPLEN, CA, USA) and Qubit2.0 Fluorometer (Life Technologies, CA, USA), followed by preparation and sequencing of RNA libraries by Metware Biotechnology Co. Ltd. (Wuhan, China) using Illumina RNA-Seq technology. Gene function annotation was performed based on NR, Pfam, KOG/COG, Swiss-Prot, KEGG and GO databases. We evaluated correlation and repetitiveness among samples using PCA and Pearson correlation coefficients, respectively. Differential expression analysis between two bamboo sheath samples was conducted using DESeq2 R package (1.16.1), with differentially expressed genes identified based on corrected *P-value* ≤ 0.05 and absolute fold change ≥ 2.

### Quantitative real-time PCR (qRT-PCR) analysis

We used quantitative real-time reverse transcription PCR to confirm RNA-Seq data reliability. The qRT-PCR analysis was conducted using Applied Biosystems ABI Quantstudio 7 Flex real-time PCR system with a 96-well plate. Vazyme’s SYBR Mixture with ROX reference dye (Nanjing, China) was used for all PCR reactions as per the instructions. We employed the Tonoplasm Intrinsic Protein 41(*TIP41*) gene as an internal control [24]. Primer pairs were designed for each gene using Primer 5.0 software, and we identified genes involved in anthocyanin biosynthesis and regulatory genes such as *C4H*, *CHS*, *CHI*, *F3H*, *DFR*, *ANS*, *UFGT*, *R2R3*-*MYBs*, *bHLH077* and *bHLH106*. Three biological samples with three replicates each were analyzed by qRT-PCR to obtain data on the relative expression frequency of target genes calculated through the 2^−△△CT^ method. Table S2 lists the primers used for quantification of gene expression by qRT-PCR.

### Statistical analysis

We performed statistical analysis on the bamboo sheath samples using Excel 2019 and Student’s *t-*tests (*P* < 0.05). We determined statistical significance with IBM SPSS statistical software 23.0 (SPSS Inc., USA) through one-way analysis of variance (ANOVA). The data were presented as mean ± standard error (SE). We used GraphPad Prism 8 to create column charts and R: pheatmap to create gene expression heatmaps.

## Results

### Anthocyanin content in three bamboo cultivars

**Fig. 1:**
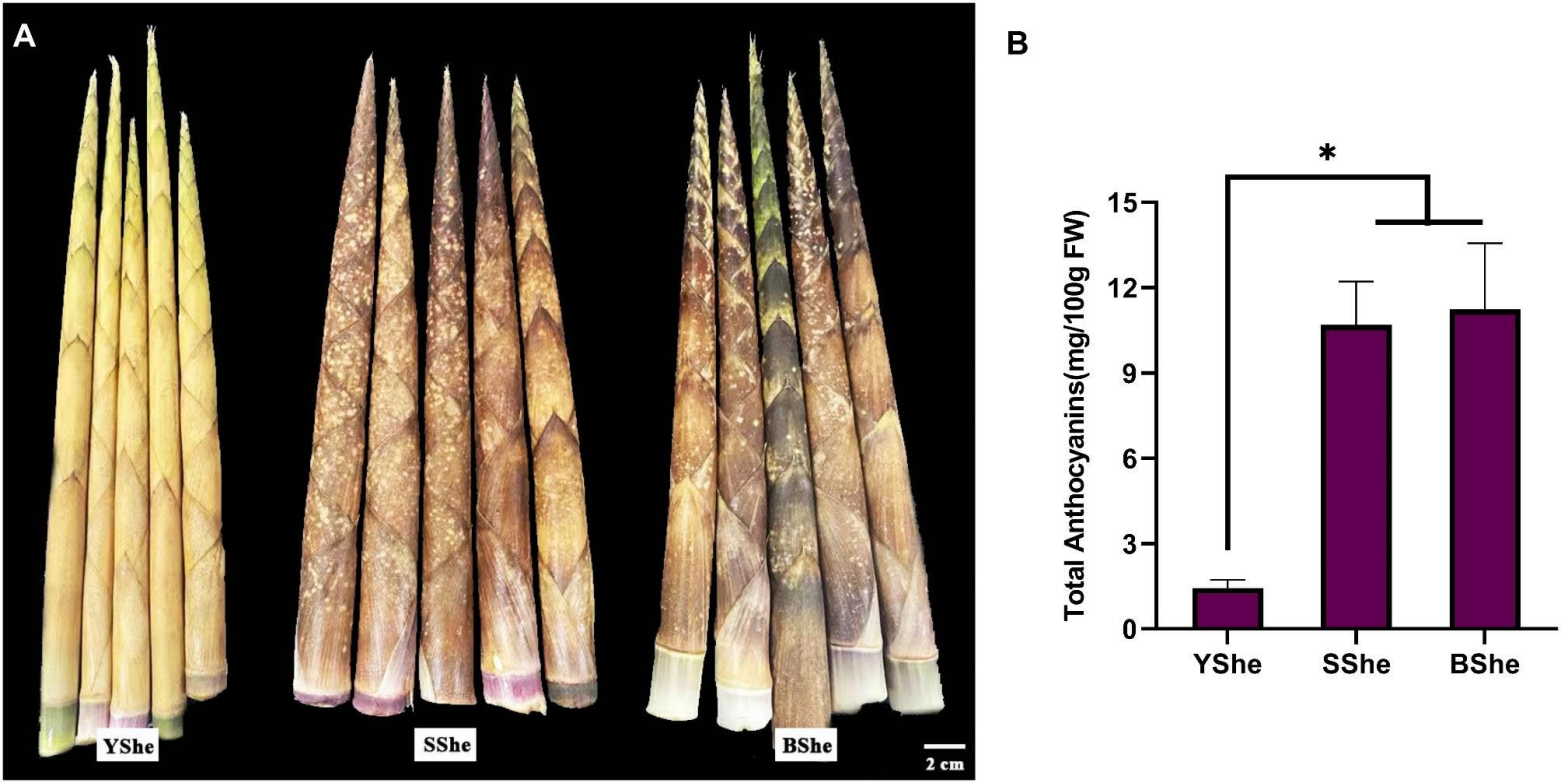
**(A)** Phenotypes **(B)** total anthocyanin content of three *C.hejiangensis* cultivars. Error bars represent standard error (SE). *Indicates *p <* 0.05

We focused on elucidating the pigment biosynthesis mechanism in *C. hejiangensis* cultivars YShe, SShe and BShe. In a visual examination of bamboo species, SShe and BShe had more purple pigment than YShe (Fig. 1A). Anthocyanin accumulation was highest in BShe (11.23 mg/100 g of FW), and SShe (10.69 mg/100 g of FW) contained more anthocyanins than YShe (1.43 mg/100 g of FW) (Fig. 1B). These results indicate that breed specificity was the main reason for the significant variation in anthocyanin accumulation.

### Quantitative metabolomic analysis of YShe, SShe, and BShe based on UPLC-MS/MS

**Fig. 2:**
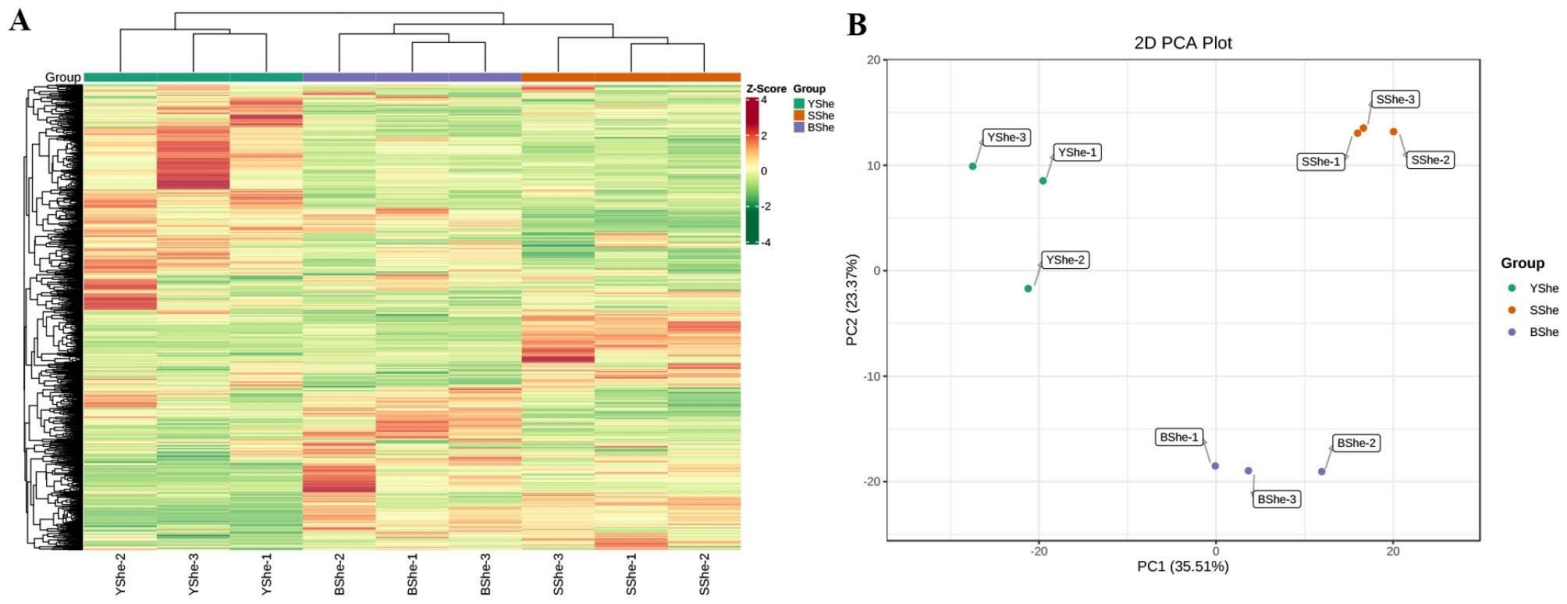
Qualitative and quantitative analysis of the metabolomics data of three *C.hejiangensis* cultivars. **(A)** Heatmap of quantitatively identified metabolites. The color scale indicates the degree of metabolite accumulation. The metabolite clustering line is shown on the left side of the figure, and the sample clustering line is shown on the top of the figure. **(B)** YShe, SShe and BShe principal component analysis

To investigate metabolite differences between bamboo sheath cultivars of different colors, we identified YShe, SShe, and BShe primary and secondary metabolites using UPLC-MS/MS analysis. The 9 samples were divided into 3 distinct groups based on clustering and correlation analysis (Fig. 2A). All samples within each group were significantly correlated (Fig. S1), and PCA demonstrated a clear separation between YShe, SShe, and BShe, indicating that their metabolic profiles differed significantly (Fig. 2B).

We identified 969 metabolites, belonging to more than 11 different classes. However, > 70% of these metabolites can be grouped into seven main classes: flavonoids (23.94%), phenolic acids (16.41%), lipids (14.96%), amino acids and derivatives (9.8%), organic acids (7.12%), alkaloids (7.02%), and lignans and coumarins (3.3%). Flavonoids were the main metabolites and can be divided into 81 flavones, 49 flavonols, 47 flavonoid carbonosides, 20 flavanones, 10 isoflavones, 8 flavanonols, 7 chalcones, 6 flavanols and 4 anthocyanidins (Table S1).

### Screening, functional annotation, and enrichment analysis of differential metabolites in three bamboo cultivars

We utilized the OPLS-DA model to compare metabolite content between YShe and SShe (R^2^X = 0.649, R^2^Y = 1, Q^2^ = 0.948), YShe and BShe (R^2^X = 0.621, R^2^Y = 1, Q^2^ = 0.911), and SShe and BShe (R^2^X = 0.628, R^2^Y = 1, Q^2^ = 0.904) to assess species differences (Fig. S2). All comparison groups had stable models with Q^2^ values above 0.9 indicating their reliability. The OPLS-DA score map demonstrated that the three varieties were separated in pairs, indicating that the metabolic phenotypes of the three varieties were significantly different.

**Fig. 3:**
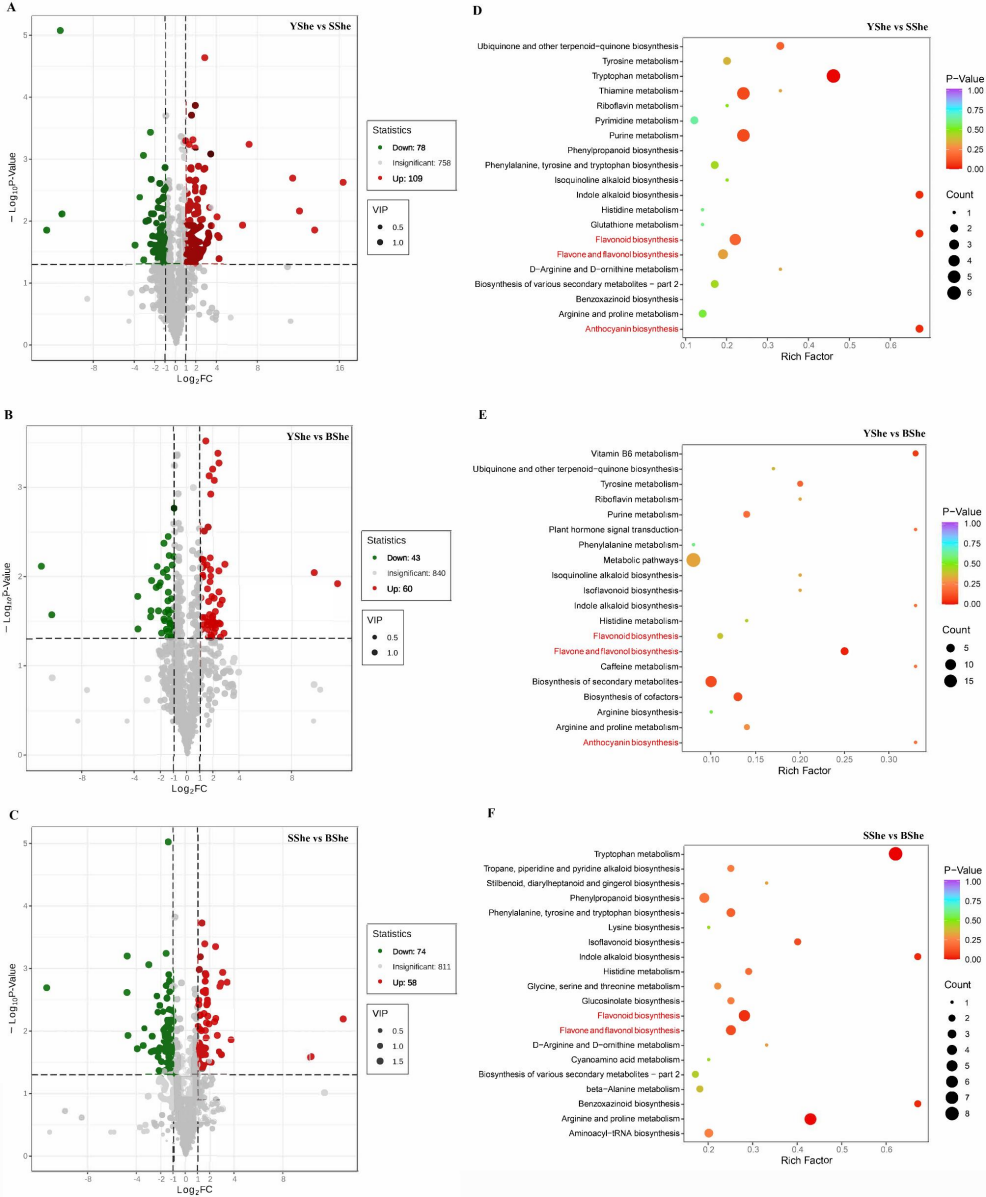
Identification and functional characterization of the differentially accumulated metabolites (DAMs) in three *C.hejiangensis* cultivars. **(A-C)** Volcano plots showing up-regulated, down-regulated and un-regulated metabolites in YShe, SShe and BShe samples. **(D-F)** KEGG enrichment analysis of DAMs between: YShe vs. SShe, YShe vs. BShe, and SShe vs. BShe. Each bubble represents a metabolic pathway with the horizontal axis and bubble size being identical. Display factors affect path length path. The bigger the bubble, the higher the hit rate. Bubble color indicates the enrichment analysis *p*-value, with darker colors indicating higher degrees of enrichment

Between YShe and SShe, there were 187 significantly different metabolites, with 109 metabolites higher in SShe. Additionally, there were 103 significantly different metabolites between YShe and BShe (60 significantly accumulated in BShe), as well as 132 significantly different metabolites between SShe and BShe (58 metabolites were higher in SShe and 74 significantly accumulated in BShe) (Fig. 3A-C). YShe and SShe differential accumulation of metabolites (DAMs) can be divided into more than 10 categories, and most are divided into 4 categories: flavonoids (49.73%), phenolic acids (13.37%), lipids (10.70%) and alkaloids (8.56%) (Fig. S3). These flavonoids were further decomposed into 28 flavones, 26 flavonoid carbonosides, 23 flavonols, 5 flavanones, 3 flavanonols, 3 anthocyanidins and 5 other flavonoids. Moreover, YShe and BShe DAMs can be grouped into similar categories with a high percentage of flavonoids (46.60%), phenolic acids (22.33%), and alkaloids (6.80%). Specifically, there were 48 flavonoids identified, including 22 flavonols, 13 flavonoid carbonosides, 7 flavones, one anthocyanidin, and 5 other flavonoid types. SShe vs. BShe DAMs included flavonoids (40.15%), alkaloids (15.91%), phenolic acids (11.36%), and lignans and coumarins (8.33%). These flavonoids could be divided into 24 flavones, 13 flavonoid carbonoside, 7 flavonols, 5 flavanones and 4 other flavonoids. The above results indicated significant differences in these metabolites, but flavonoids were identified as the main differential metabolites for variations in YShe, SShe, and BShe.

Additionally, 83 out of 93 flavonoids that differentially accumulated between YShe and SShe were higher in SShe, while 43 out of 48 flavonoids that differentially accumulated between YShe and BShe were higher in BShe (Table S1). A total of 30 flavonoids accumulated in both SShe and BShe according to Venn diagram analysis. These include apigenin-6-C-xyloside-8-C-arabinoside, carlinoside, syringetin-3-O-(6’’-acetyl)glucoside, limocitrin-3-O-glucoside, okanin-4’-(6’’-O-acetyl)glucoside, narcissin, hesperetin-5-O-glucoside, cyanidin-3-O-rutinoside, and other DAMs (Fig. S4). These compounds are related to phenylpropanoid biosynthesis, flavonoid biosynthesis, flavone and flavonol biosynthesis, and anthocyanin biosynthesis, serving as intermediates and end products. It is speculated that the differences in flavonoid accumulation may be attributed to variations in cultivars and genetic specificity.

We matched all control group DAMs with the KEGG database to identify the metabolic pathways involving these metabolites. We performed enrichment analysis on the annotation results to obtain pathways highly enriched in DAMs. The KEGG terms enriched in DAMS detected in all comparative samples were secondary metabolite biosynthesis, phenylpropanoid biosynthesis, flavonoid biosynthesis, flavone and flavonol biosynthesis and anthocyanin biosynthesis (Fig. 3D–F). The diverse potential metabolites suggest that nutrients and antioxidants may be related to bamboo sheath color changes. Phenylpropanoid and flavonoid-related molecules were enriched in these unclear conserved metabolites. Based on the role of flavonoids in plant coloring, we infer that DAMs from the flavonoid biosynthesis pathway may be the key metabolites for bamboo sheath color changes.

### RNA-Seq analysis of three bamboo cultivar sheaths

**Fig. 4:**
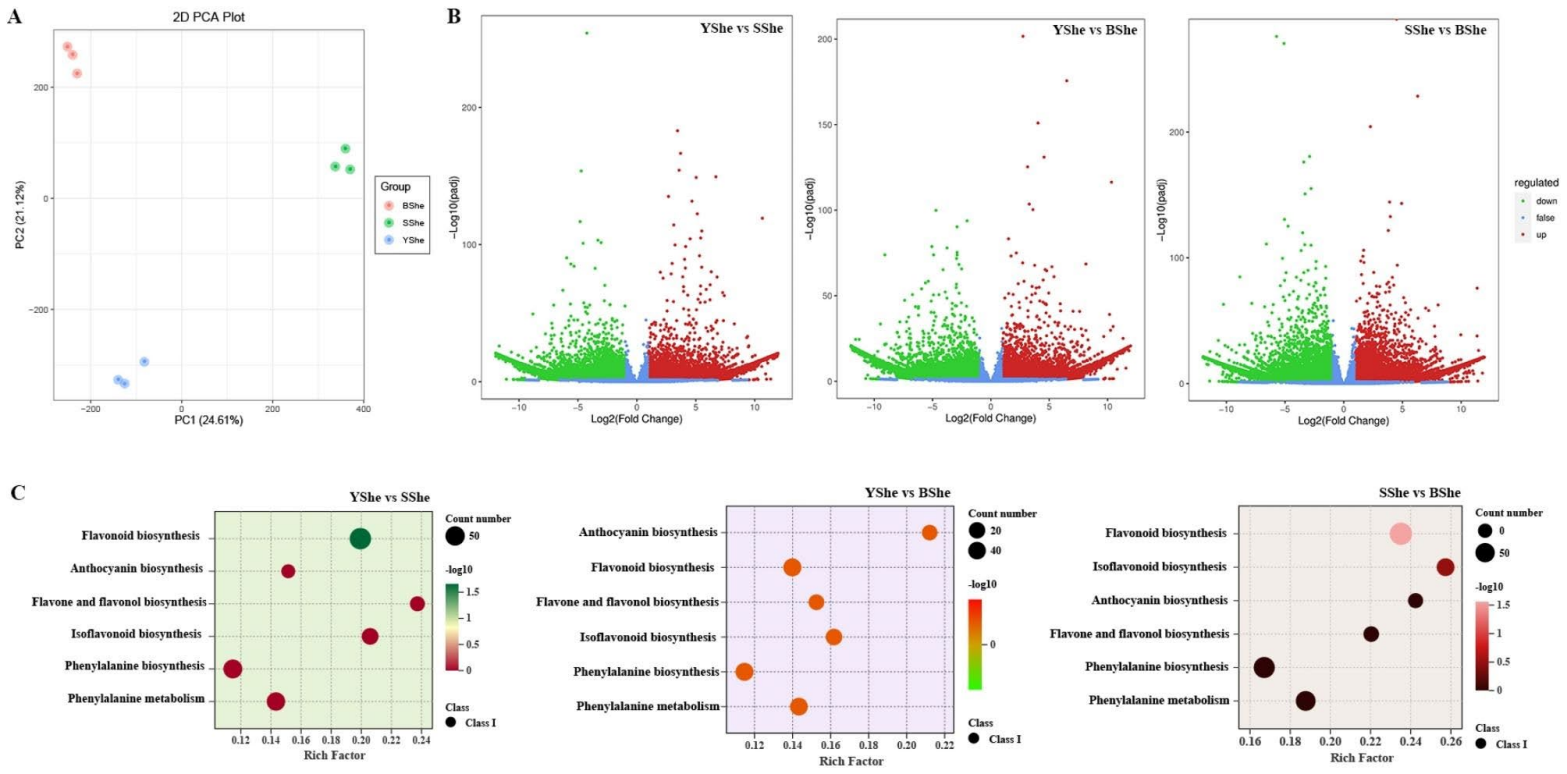
Differential expressed genes (DEGs) in three *C.hejiangensis* cultivars. **(A)** YShe, SShe and BShe PCA analysis. **(B)** Volcano plots of up-, down-, and down-regulated genes expressed in YShe, SShe, and BShe samples. **(C)** KEGG pathway enrichment statistics. Dot size represents recording frequency

We extracted and analyzed three cDNA libraries (YShe, SShe, and BShe) to investigate the anthocyanin accumulation mechanism in bamboo sheaths. We used RNA-seq on the Illumina HiSeq 2000 platform with three biological replicates for each of the nine samples. We obtained 56.38 GB of clean data, with at least 5.86 GB per sample. The Q30 base percentage was 91.92% or higher in each sample (Table S3). In total, we collected 334,559 unigenes from the three libraries and annotated them using GO, KO, KOG, NR, NT, PFAM and SwissProt databases. The sequencing data was highly reliable based on the clustering of all biological replicates in the Principal Component Analysis (PCA) (Fig. 4A). PC1, which accounted for 24.61% of the data variance, represents differences between YShe, SShe, and BShe samples. Additionally, there was a clear separation between samples of different colors as also seen in metabolomic analysis. This suggests that differences in gene expression tightly control changes in metabolite accumulation.

### Differential expression of flavonoid structural and regulatory genes

We calculated FPKM values for gene expression profiling and used them to normalize expression in each sample. Totally 26,043 DEGs between YShe and SShe were identified with an FDR ≤ 0.05 and FC ≥ 1. We identified 12,345 up-regulated genes and 13,698 down-regulated genes in YShe. Compared to BShe, YShe exhibited an upregulation of 11,174 genes and a downregulation of 11,480 genes (Fig. 4B). To further investigate DEGs across all three bamboo species, we conducted KEGG enrichment analysis. Given our metabolomic findings, we specifically examined the flavonoid pathways. Phenylpropanoid biosynthesis (ko00940) was the most abundant, followed by flavonoid biosynthesis (ko0941) (Fig. 4C).

**Fig. 5:**
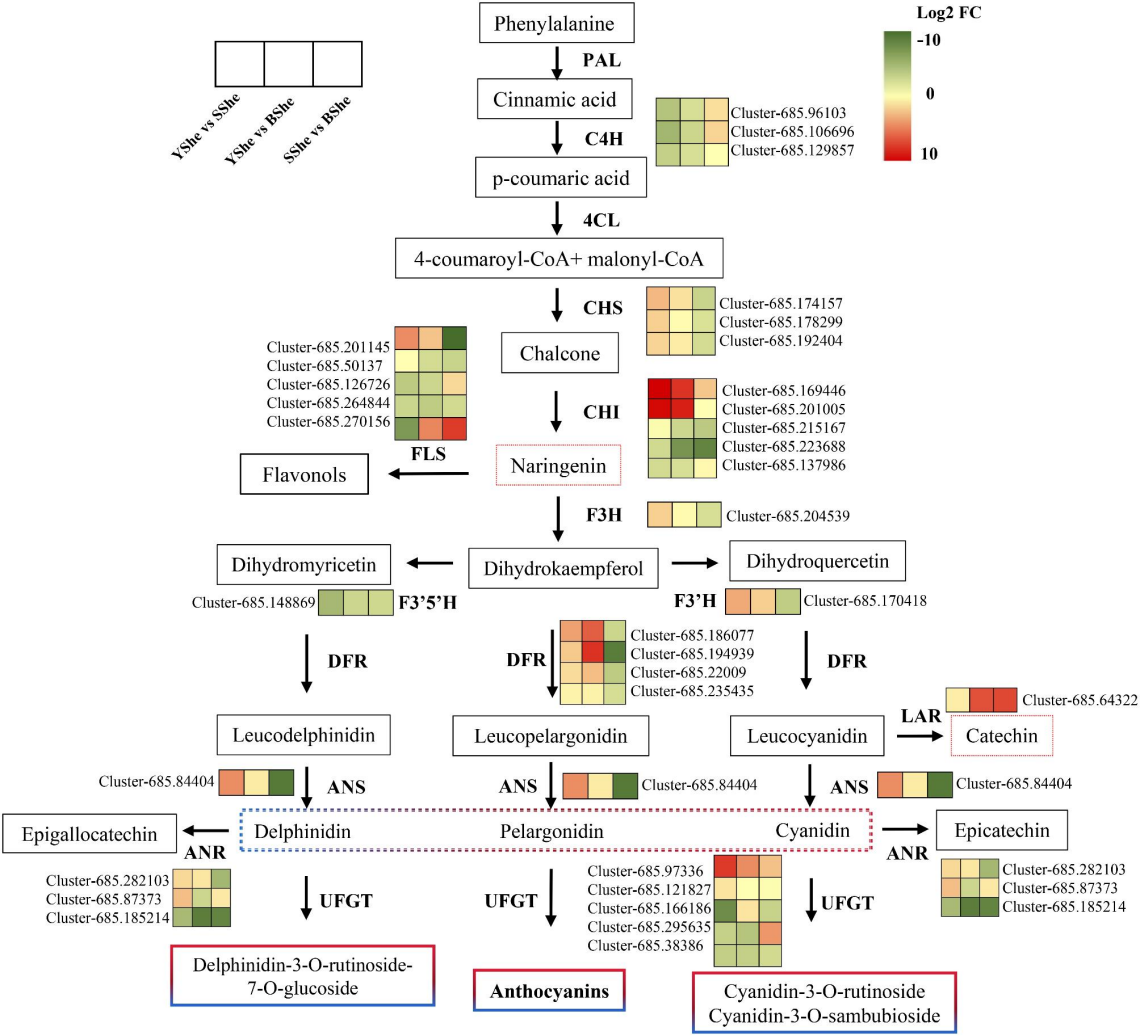
A putative transcriptional regulatory network as a metabolic pathway for flavonoid biosynthesis. The anthocyanin biosynthesis pathway was drawn based on differentially expressed structural genes in flavonoid metabolism. The heatmap was generated from Log2FC values of the transcriptome dataset, with columns and rows representing samples and genes, respectively. This figure (KEGG: map00941 and map00942) was downloaded from the KEGG website with copy-right permission

Because we focused on differences in anthocyanin accumulation in different bamboo species, we identified the expression of each gene encoding a protein involved in anthocyanin biosynthesis. YShe exhibited similar differential expression patterns of flavonoid structural genes as SShe and BShe. It contained three *C4H*, three *CHS*, five *CHI*, one *F3H*, one *F3′H*, one *F3′5′H*, five *FLS*, four *DFR*, one *LAR*, three *ANR*, one *ANS*, and five *UFGT* gene(s) (Fig. 5). Notably, anthocyanin biosynthesis genes that increased to varying degrees accounted for the majority, suggesting a possible link between them and the colors of various bamboo species. This information is valuable for studying how anthocyanin accumulates in bamboo sheaths at a molecular level.

Transcription factors, including *bHLH*, *MYB*, and *WD40*, are crucial in regulating flavonoid biosynthesis. They activate anthocyanin biosynthesis genes alongside structural genes [25]. Between YShe and SShe, 166 flavonoid regulatory genes were differentially expressed (Table S4). Seventy genes were upregulated in SShe, including 20 *bHLH* genes, 17 *MYB* genes and 33 *WD40* genes. There were 73 up-regulated genes between YShe and BShe, including 13 *bHLH* genes, 26 *MYB* genes, and 34 *WD40* genes. Likewise, up-regulated expression of 133 flavonoid regulatory genes was detected between SShe and BShe, including 43 *bHLH* genes, 68 *MYB* genes, and 22 *WD40* genes (Table S4). Significant differences in the expression of *MBW* transcription factors were found among the three bamboo species, which could result in variations in anthocyanin levels and subsequent color differences.

### UPLC–MS and RNA-Seq profile combined analysis

**Fig. 6:**
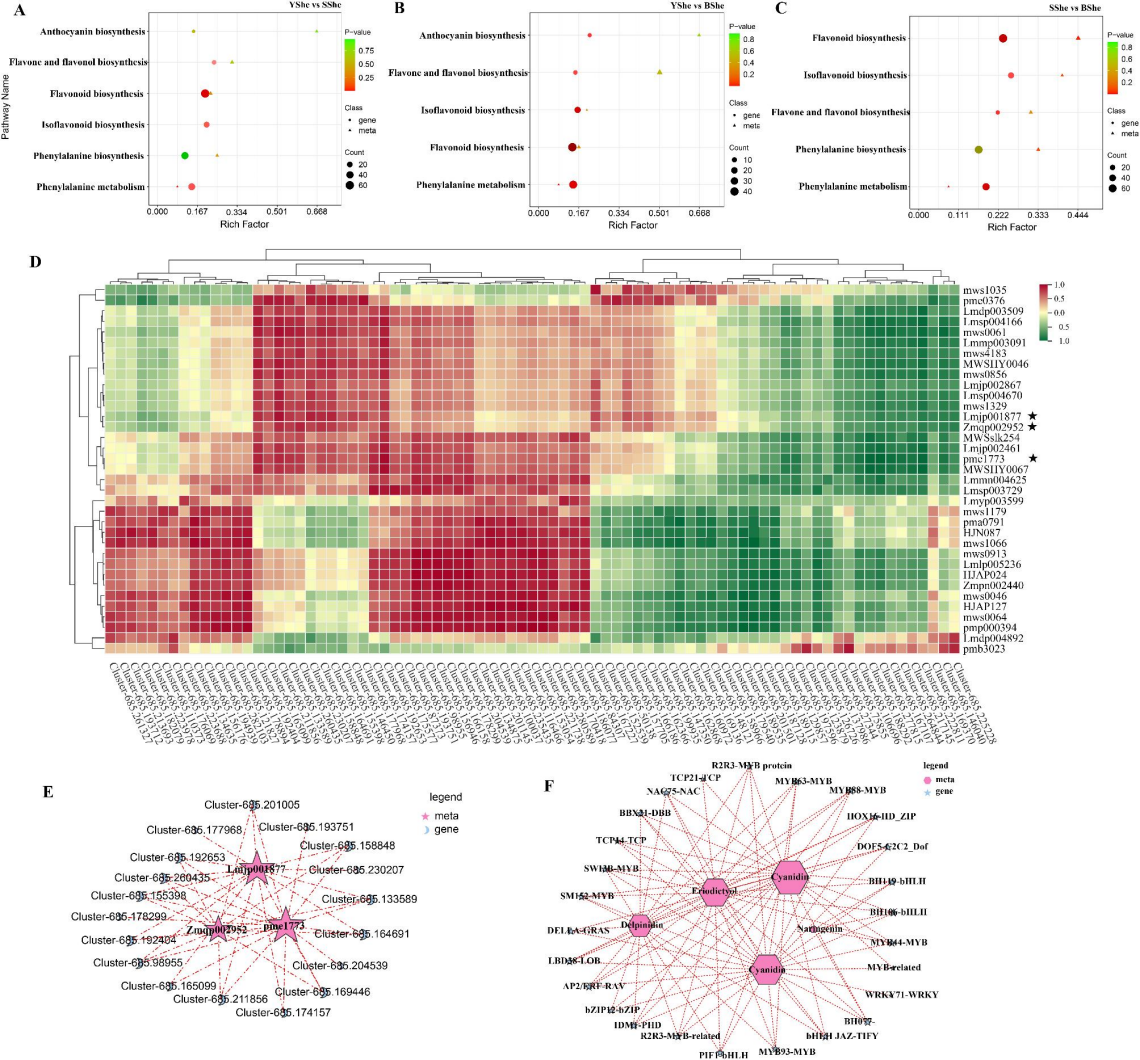
Regulatory network of anthocyanin-related compounds in bamboo sheaths. **(A-C)** Scatter plot showing the accumulation of DEGs and DAMs in the flavonoid biosynthetic pathway using the KEGG pathway. Genes are represented by circles and metabolites by triangles. The comparison is between YShe and SShe, YShe vs. BShe, SShe vs. BShe. **(D)** Correlation analysis diagram depicting the relationship between DEGs and DAMs in flavonoids metabolic pathways. Each row represents a DAM, while each column corresponds to a DEG. The colors reflect changes in correlation coefficient: red represents correlation coefficients with high and positive correlation and green indicates high and negative correlation (legend presents detailed correlation coefficient). **(E-F)** Co-expression network plots of anthocyanin-related compounds and genes. **(E)** anthocyanin metabolites associated with structural genes. **(F)** Anthocyanin metabolite connections with transcriptional regulatory genes

We investigated the relationship between transcriptome and metabolome in bamboo sheaths by DEGs and DAMs connections analysis. Combined with KEGG pathway enrichment results (Fig. 6A-C), it revealed significant enrichment of DEGs and DAMs involved in phenylalanine biosynthesis, phenylalanine metabolism, flavone and flavonol biosynthesis, flavonoid, isoflavonoid and anthocyanin biosynthesis across three bamboo species. This suggests that changes in metabolite accumulation are closely linked to differences in gene expression. Notably, we found a significant association between DAMs and DEGs involved in the flavonoid synthesis pathway. To further explore this relationship, we calculated *Pearson’s* correlation coefficients between DAMs and DEGs within this pathway using log2FC values for each metabolite and transcript. *R*^*2*^ > 0.8 and *p* < 0.05 were chosen as the significance levels (Fig. 6D). In total, 36 flavonoid synthesis-related metabolites and 94 flavonoid synthesis-related genes were analyzed. We focused on three metabolites of the anthocyanin pathway that are strongly associated with 18 structural genes (Fig. 6E), including *C4H* (Cluster-685.96103, Cluster-685.106696, Cluster-685.129857) genes, *CHS* (Cluster-685.174157, Cluster-685.178299) genes, *CHI* (Cluster-685.169446, Cluster-685.201005) genes and *DFR* (Cluster-685.186077, Cluster-685.194939) genes. These encode key enzymes in anthocyanin biosynthesis. We constructed a co-expression regulatory network of 29 transcription factors related to the anthocyanin pathway, such as *MBW* complex, *bZIP*, *TCP*, *ERF* and *WRKY*. Their expression was highly correlated (*r* > 0.8) with five typical metabolites (Fig. 6F), and these regulatory genes may control anthocyanin biosynthesis in colored bamboo sheaths.

**Fig. 7:**
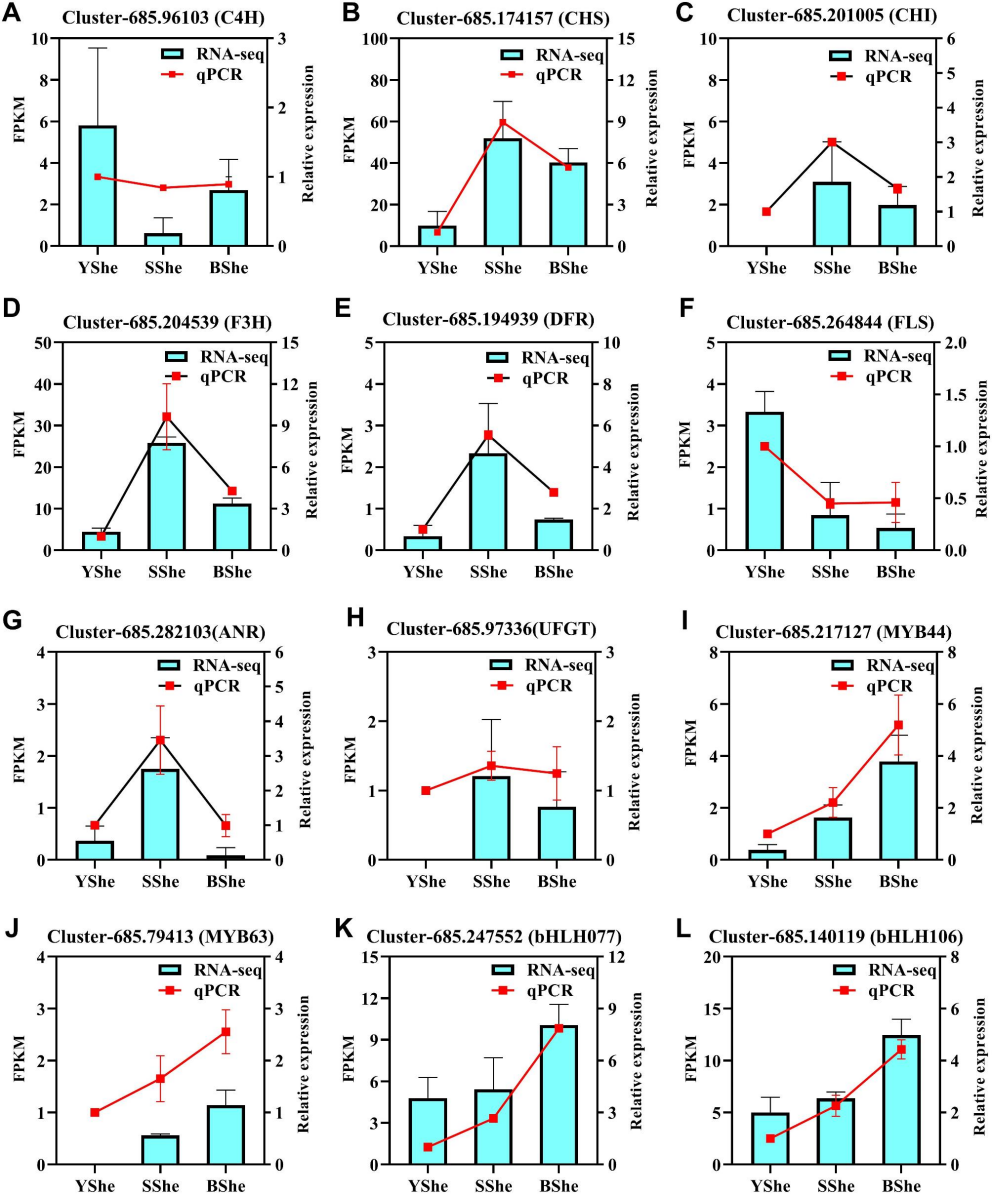
RT-qPCR verification of expression patterns of transcriptome data. (**A-H**) Eight structural genes of the flavonoid biosynthesis pathway. (**I-L**) Four transcription factor genes involved in the regulation of flavonoid biosynthesis. Vertical bars represent means ± standard deviations (SD)

We validated the transcriptome analysis results of 8 flavonoid structural genes and 4 flavonoid regulated genes using qRT-PCR. The expression patterns of all 12 genes were consistent with the RNA-Seq data (Fig. 7), and *Pearson’s* correlation coefficients indicated a strong agreement between numerical measures of transcript abundance and qRT-PCR data for most genes. These findings confirm the reliability of RNA-seq data and support its application to transcriptome analysis.

## Discussion

### Metabolites identified in the three bamboo cultivar sheaths

We provide a detailed analysis of the primary and secondary metabolites found in bamboo sheaths, reporting a total of 969 metabolites. We identified flavonoids, phenolic acids, lipids, amino acids and derivatives, organic acids and alkaloids as the main components of bamboo sheaths. This finding differs from previous studies that reported limited types of bioactive substances. Our results significantly contribute to our understanding of the chemical composition of bamboo sheaths. Previous research has identified flavonoids as the main antioxidant constituents of bamboo leaves [26]. Wang et al. used liquid chromatography and mass spectrometry to identify 97 flavonoid monomers in bamboo leaves and shoots, with most being glycosides [27]. For bamboo sheath samples, we identified 232 specific flavonoid monomers comprising various types such as flavones (81), flavonols (49), flavonoid carbonoside (47), flavanones (20), isoflavones (10), flavanonols (8), chalcones (7), flavanols (6) and anthocyanidins (4) (Table S1). We identified 187 tentatively flavonoids in SShe, which is consistent with previous research. Additionally, our DAMs analysis indicated that SShe has a higher antioxidant content than YShe (Fig. 3).

Anthocyanins are known to have excellent antioxidant ability [28, 29]. Purple-sheath bamboo cultivars had a higher anthocyanin content compared to yellow-sheath bamboo, and there was a significant variation in anthocyanin content among different varieties (Fig. 1B). We detected anthocyanins in YShe, SShe and BShe bamboo sheaths by UPLC-MS/MS analysis. Bamboo sheaths contained four types of anthocyanins: malvidin-3-O-glucoside, cyanidin-3-O-sambubioside, cyanidin-3-O-rutinoside, and delphinidin-3-O-rutinoside-7-O-glucoside (Table S1). Notably, malvidin content was significantly higher in YShe than in BShe, indicating that YShe also accumulates anthocyanins, which is consistent with the measured anthocyanin content. The three bamboo species contained the same types of anthocyanins, but with varying concentrations, suggesting possible differences in anthocyanin biosynthesis or regulatory gene expression among them.

### TFs regulate the expression of flavonoid/anthocyanin structural genes

We identified four R2R3-*MYB* transcription factors (cluster-685.217127, *MYB44*; cluster-685.79413, *MYB63*; cluster-685.85150, *MYB88*; cluster-685.115668, *MYB93*), three *bHLH* transcription factors (cluster-685.247552, *bHLH077*; cluster-685.140119, *bHLH106*; cluster-685.163830, *bHLH119*), and two *WD40* (cluster-685.170153, cluster-685.171320) repeat proteins as the main flavonoid/anthocyanin regulatory factors in bamboo sheaths. These findings are supported by previous research on the formation of the *MBW* complex [30] and its involvement in regulating structural genes for anthocyanin biosynthesis [31] (see Table S4 and Fig. 6 for details). Therefore, we speculate that the MBW complex, consisting of *MYB*, *bHLH* and *WD40* transcription factors, plays a crucial role in regulating anthocyanin accumulation in bamboo sheaths.

Other than the MBW complex, various transcription factors indirectly regulate anthocyanin biosynthesis. Several transcription factor families such as bZIP bZIP [32], TCP [33], ERF [34], WRKY [35], DBB [36] and SBP [37] play a role in regulating anthocyanin accumulation. We also found significant differences in the expression levels of *bZIP* (cluster-685.170171, cluster-685.127753), *TCP* (cluster-685.195325, cluster-685.227000), *ERF* (cluster-685.110270, cluster-685.176510), *WRKY* (cluster-685.165628), *BBX21* (cluster-685.199868) and *SPL* (cluster-685.261018) family genes among SShe and BShe bamboo sheaths, which suggests their potential role in regulating flavonoid/anthocyanin synthesis in these tissues. Further research is necessary to understand their function in flavonoid biosynthesis.

## Conclusions

We investigated the mechanism of bamboo sheath color variation among three *C.hejiangensis* cultivars using metabolomics and transcriptomics approaches. We identified 969 metabolites, including 232 flavonoid metabolites, through UPLC-MS analysis. Flavonoids were the major differential metabolites that determined bamboo sheath coloration. A comparison between purple-sheath (SShe/BShe) and yellow-sheath cultivars (YShe) revealed that about 49.73% and 46.6% of flavonoid DAMs were identified, respectively. Combined metabolomic and transcriptomic analyses helped identify various structural gene regulatory patterns involved in flavonoid biosynthetic pathways. These findings provide valuable insights into the *C.hejiangensis* color variation mechanism and offer a list of candidate metabolites/genes for genetic breeding purposes to develop flavonoid-rich cultivars.

### Electronic supplementary material

Below is the link to the electronic supplementary material.

Supplementary Material 1

Supplementary Material 2

Supplementary Material 3

Supplementary Material 4

Supplementary Material 5

## Data Availability

The raw RNA-seq datasets supporting the conclusions of this article are available in the China National Center for Bioinformation (https://www.cncb.ac.cn/), accession number: PRJCA016920. Metabolomics data can be found at: https://github.com/LeiYu-Bio/Chimonobambusa-hejiangensis-Project-DATA.
