# Supplementary material for: Characterization of color variation in bamboo sheath of Chimonobambusa hejiangensis by UPLC-ESI-MS/MS and RNA sequencing
Source: BMC Plant Biol. 2023 Oct 6;23:466. doi: 10.1186/s12870-023-04494-3 (PMC10557168; doi:10.1186/s12870-023-04494-3)
Supplement: Supplementary file 4 — Supplementary Material 4 [file 12870_2023_4494_MOESM4_ESM.docx]

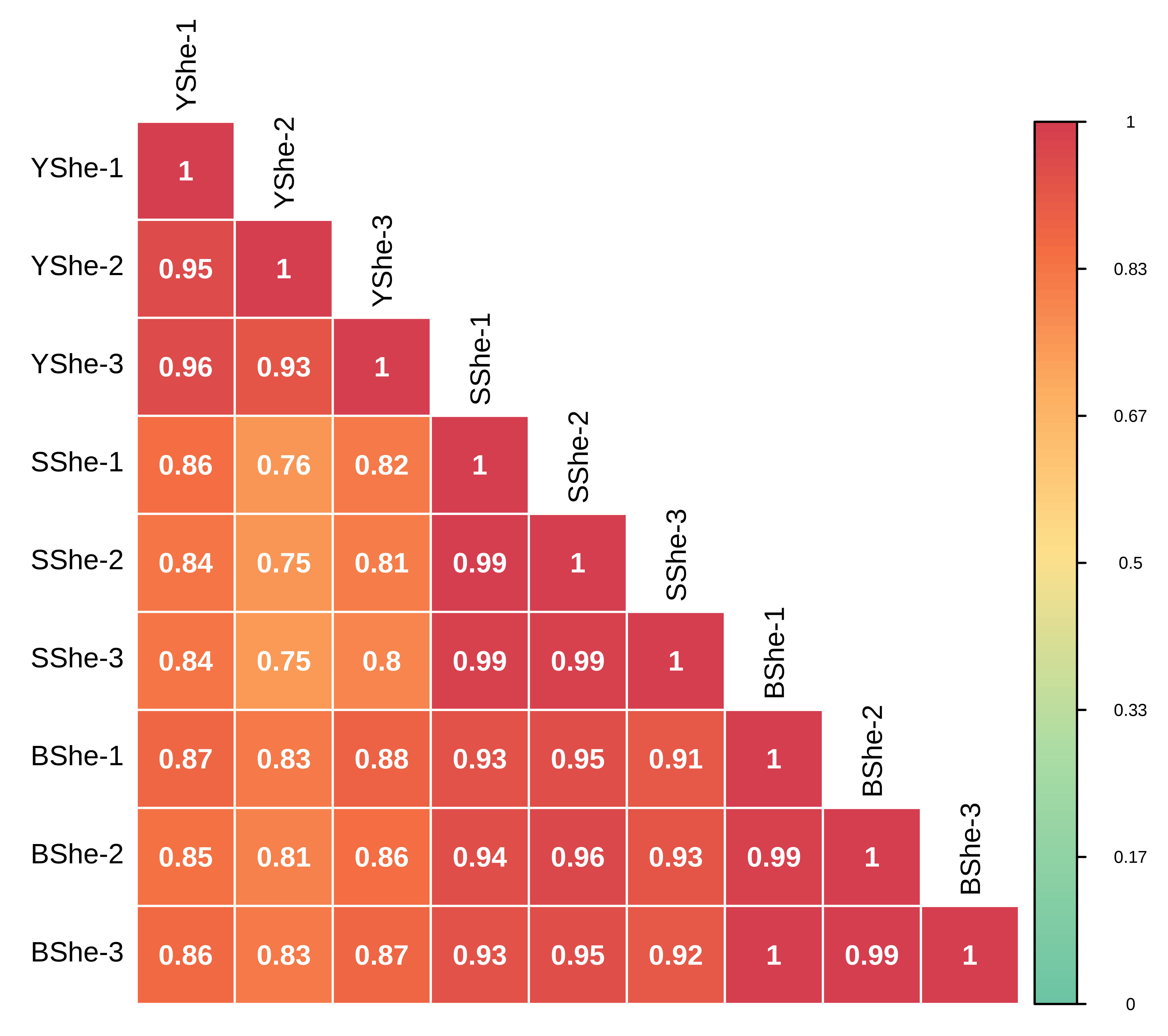


**Supplementary Figure 1.** **The intragroup samples showed significant correlations.** The vertical and diagonal lines represent the sample names of different samples, different colors represent different Pearson correlation coefficient size, the redder the color represents the stronger positive correlation, the greener the color represents the worse correlation, the bluer the color represents the stronger negative correlation, while the correlation coefficient size between two samples is marked in the square.


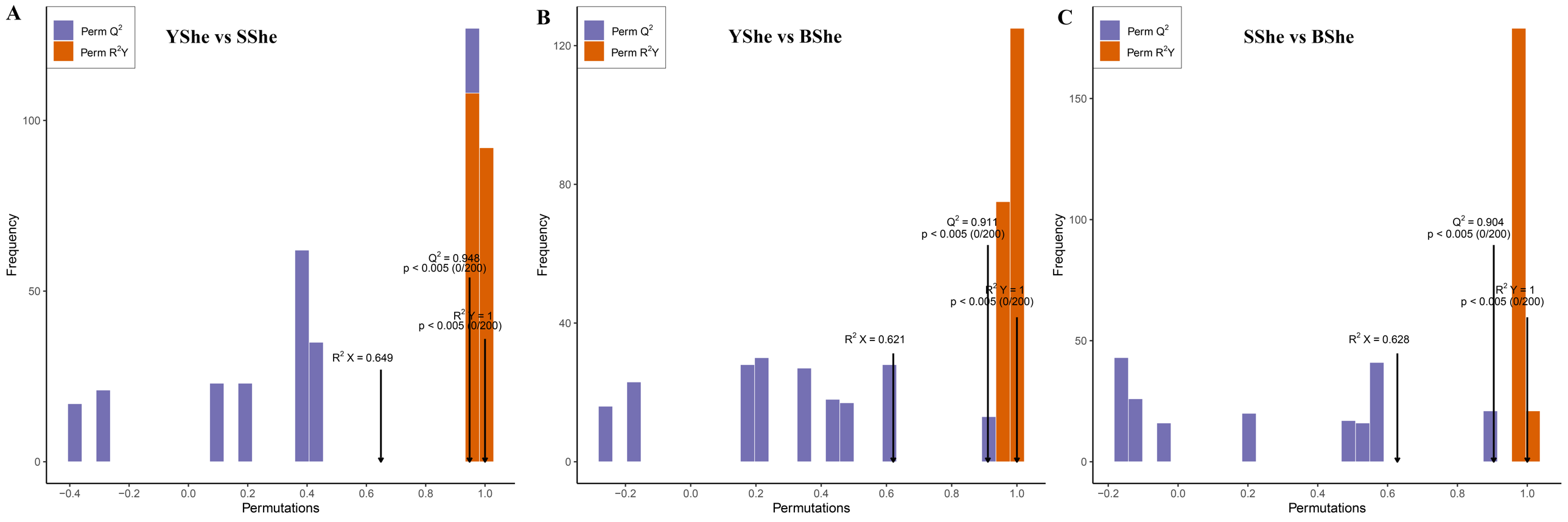


**Supplementary Figure 2.** **The OPLS-DA model compared metabolite contents of the varieties in pairs to evaluate the differences between YShe and SShe/BShe.**


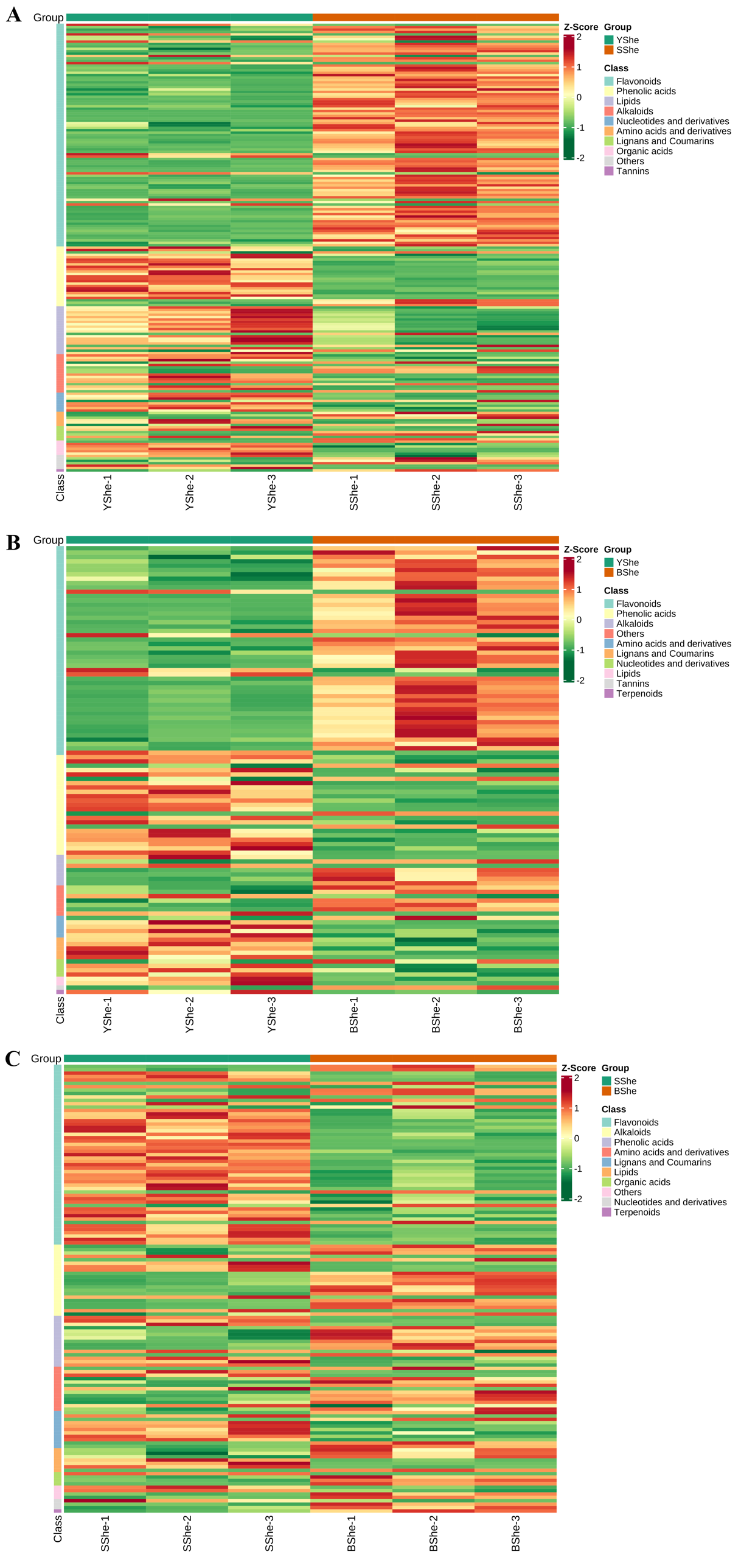


**Supplementary Figure 3.** **The differential metabolites in the different samples.** The horizontal coordinate is the sample name, the vertical coordinate is the differential metabolite, and the different colors in the heat map represent the values obtained after normalization of the relative content of the differential metabolite, reflecting its relative content (red represents high content, green represents low content), and the comment bar above the heat map corresponds to the sample group (Group); for the classification of the differential metabolite, the comment bar on the left side of the heat map corresponds to the first level of substance classification (Class), different colors represent different substance classes.

(C) Venn diagram showing the differential accumulated metabolites (DAMs) of comparison groups.


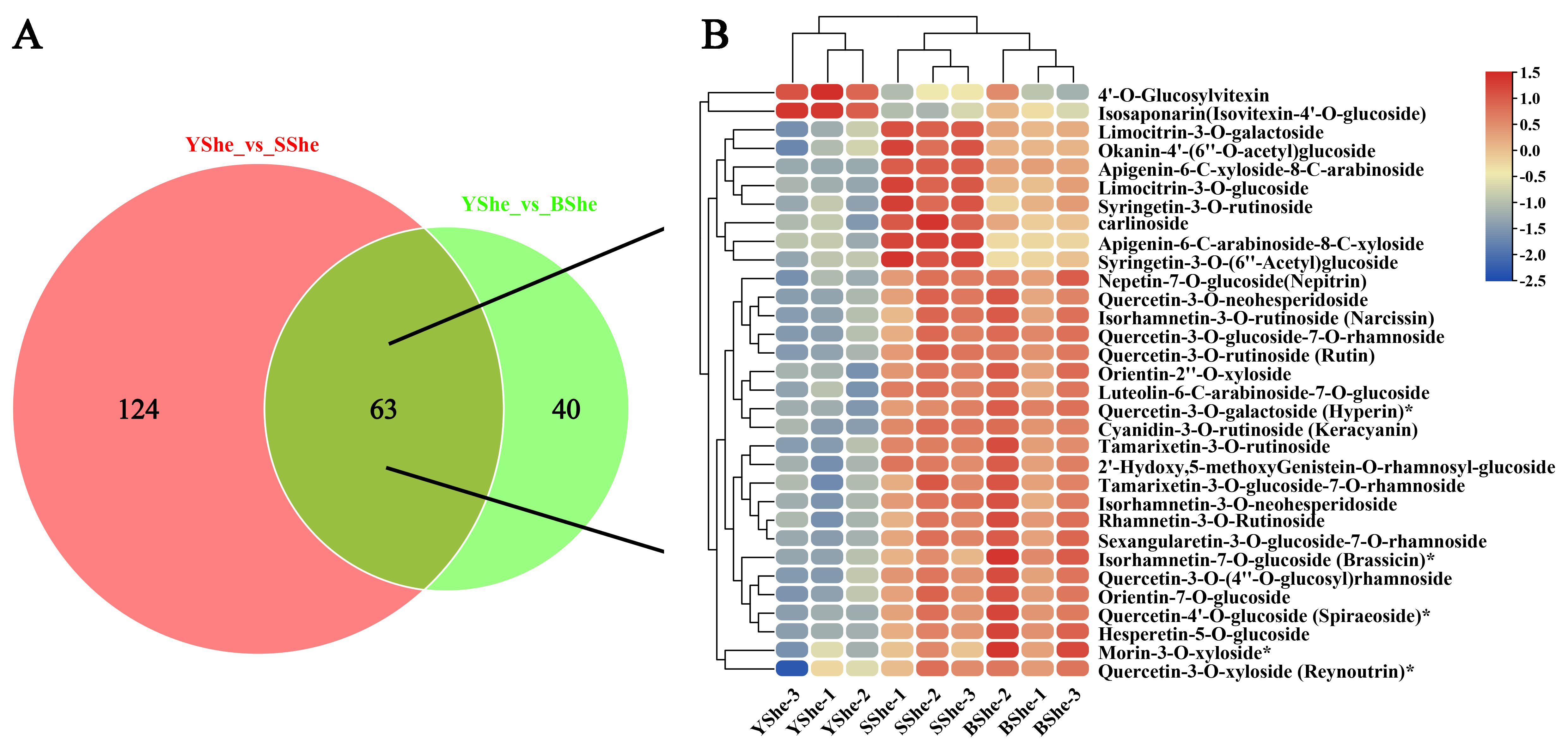
**Supplementary Figure 4.** **Analysis of flavonoid metabolites in bamboo shoot sheaths.** (A) Venn diagram depicting the shared and the specific number of flavonoids in different comparison groups. (B) Hierarchical clustering of flavonoids in all datasets. Detailed samples are shown in Table S1.
